# Mapping Rare Erythrocyte Phenotypes in Morocco: A Tool to Overcome Transfusion Challenges

**DOI:** 10.1155/2014/707152

**Published:** 2014-03-12

**Authors:** A. Benahadi, S. Boulahdid, B. Adouani, A. Laouina, A. Mokhtari, A. Soulaymani, K. Hajjout, M. Benajiba, R. Alami

**Affiliations:** ^1^Centre National de Transfusion Sanguine, Rue Lamfadal Charkaoui, Madinat Al Irfane, BP 180, Rabat, Morocco; ^2^Laboratoire de Génétique et de Biométrie, Faculté des Sciences, University Ibn Tofaïl, Kenitra, Morocco

## Abstract

The aim of this research is to search for the distribution of blood groups in all the regions of Morocco. This study, done for the first time, aimed to provide the frequency of the Rhesus system and Kell (K) in more than 55000 blood donors from nine different regions around the country. In addition, the frequency of the Cellano, Duffy, Kidd, and MNS blood antigens was searched for 500 blood donors from the Rabat's region. Frequency of blood donors with rare blood groups was characterized for the first time in the country and compared to results found from other populations.

## 1. Introduction

Blood group phenotypes have been used for several applications as for blood transfusion practices and population genetic studies [1–3].

In Morocco, no study has identified yet the distribution and frequency of different blood groups. In this perspective we tried to identify the Rhesus phenotypes in nine different regions spanning the whole country. We have also conducted a study to determine the frequencies of the k (cellano), Fya, Fyb, Jka, Jkb, S, and s antigens in blood donors (BD) from the Rabat region.

## 2. Materials and Methods

Fifty-five thousand six hundred and thirty (*N* = 55630) BD who gave blood in nine different regions were phenotyped for the following Rhesus blood antigen D, C, E, c, and e. In addition, from the CRTS Rabat, we phenotyped 513 BD in the S and s antigens from the MNS system, Fya and Fyb antigens from the Duffy system, Jka and Jkb antigens from the Kidd system, and k (cellano) antigen from the Kell system.

For the determination of Rhesus and Kell typing, we used the OLYMPUS PK7300 Automated System and/or microplates by standard hemagglutination test using commercial monoclonal antisera IgM anti-D, anti-C, anti-c, anti-E, anti-e, and anti-K monoclonal antibodies (Diagast). All samples that showed negative agglutination with monoclonal/polyclonal IgM/IgG anti-D and the Fy (a−, b−) phenotype were confirmed using Coombs' test. Fya, Fyb, Jka, Jkb, S, s, and k (cellano) antigens were typed by commercially prepared polyclonal antisera (Seraclone) with microplates methods for S antigen and by hemagglutination in gel cards (BIO-RAD,ORTHO/BLISS, INVITROGEN) for other antigens.

Positive and negative control cells and Coombs' control cells were used for quality controls.

The allele frequencies were calculated using the gene counting method, which was described by Mourant et al. in 1976 [4].

Statistical analysis was carried out using Microsoft Excel and the program PASTE [5].

## 3. Results and Discussion

We characterized the Rhesus antigens distribution in 55630 randomly chosen BD from 9 regions, representing different ethnic groups of Morocco.

The Rhesus antigens frequencies found were as follows: e (89.45%), D (70.65%), c (60.58%), C (38.54%), and E (9.59%). The observed phenotypes distributions are summarized in Table 1. The CcDee phenotype was represented in all the studied regions. Its frequency ranged from 34.23% in Safi to 40.67% in the south of the country at Laayoune. By contrast, three phenotypes (CcDEE, CcddEe, and ccddEE) were characterized only in Rabat BD with the lowest frequency in the country (0.0082%, 0.0062%, and 0.0041%, resp.).

Little data are available regarding the frequencies of the blood group antigens other than ABO and RhD in the Moroccan population. In this study, we examined the composition of RBC antigens in Moroccan BD. It is the first study in Morocco that involved such large number of BD (*N* = 55630). The D antigen is present in 70% of the BD population, thus showing an intermediate prevalence between those found in the African and European continents [6–8]. The e antigen (89%) and c antigen (61%) are most frequent in Morocco; this is concordant with Tagny et al.'s, 2010 [9].

The most common Rhesus haplotypes in Morocco are DCe, followed by Dce and then by dce, respectively, found at 0.38, 0.34, and 0.28. This prevalence is similar to our neighbor Algerians [10] and Tunisians [11]. While in Mauritania and in Sub-Saharan Africa, it is the Dce that shows the highest prevalence [6, 7, 12].

In addition, Principal Component Analysis showed a clear evidence of a north-to-south gradient for some Rhesus phenotypes. In fact the DCcEe, CCDee, and ccDEe are linked to the north regions (Tétouan, Oujda, and Al-Hoceima) while the Dccee is associated with the south provinces (Laayoune and Ouarzazate). This gradient was also found in a previous study by our group for the D antigen [1].

Other system (Kell, MNS, Duffy, and Kidd) phenotype frequencies showed that out of 513 Rabat's blood donors, only one donor (0.19%) was homozygote for the K. Cellano represented 99.80%. A north-to-south gradient was observed for the Kell antigen.

The S and s antigens were positive in 43.1% and 91.2%, respectively. Fy (a− b+) was the most common phenotype seen in about 42% of the studied subjects. The Duffy null or Fy (a− b−) phenotype was observed in 11.11%. This latter phenotype is referred to in the African population as discussed by different authors [7, 13, 14].

For the Kidd system, about half (49.5%) of our BD have the Jk (a+ b+), as found in the European population [15]. The Jk (a− b−) was not found in the studied population. Jka and Jkb antigens were recorded in 84.21% and 65.3% of subjects, respectively (Table 2).

## 4. Conclusion

Since the current practice of providing compatible blood to patients in Morocco is still relaying on blind cross-matching of available red blood cell units, we initiated this study to map the distribution of the blood groups around Morocco, especially for better management of red cell unit delivery to highly alloimmunized patients.

The main result found here illustrated that the Moroccan population shares phenotypes with Sub-Saharan Africa and European populations, and a clear evidence of a north-to-south gradient for some Rhesus phenotypes.

Some particularities were also found as the higher frequency of “e” antigen blood group and the Fy (a− b−) which was represented at a significant frequency (11.1%).

## Figures and Tables

**Table 1:** Rhesus phenotypes found in the different regions of Morocco.

Regions	Rabat *N* (%)	Meknès *N* (%)	Tétouan *N* (%)	Safi *N* (%)	BeniMellal *N* (%)	Al-Hoceima *N* (%)	Oujda *N* (%)	Laayoune *N* (%)	Ouarzazate *N* (%)	Total (Moroccan population) (%)
*N*	**48610**	**2138**	**1002**	**298**	**1231**	**462**	**1285**	**118**	**486**	**55630**
CcDee	18825 (38.727)	794 (37.138)	344 (34.331)	102 (34.23)	499 (40.536)	181 (39.177)	463 (36.031)	48 (40.678)	148 (37.860)	** 38.54**
ccDee	9444 (19.428)	436 (20.393)	164 (16.367)	59 (19.8)	229 (18.603)	59 (12.771)	214 (16.654)	28 (23.729)	111 (22.840)	**19.31**
CCDee	7396 (15.215)	307 (14.359)	200 (19.960)	52 (17.45)	200 (16.247)	87 (18.831)	232 (18.054)	17 (14.407)	84 (17.284)	**15.41**
ccDEe	4590 (9.442)	209 (9.775)	103 (10.279)	23 (7.718)	81 (6.580)	40 (8.658)	118 (9.1829)	8 (6.7797)	26 (5.350)	**9.34**
ccddee	3781 (7.778)	184 (8.606)	82 (8.184)	34 (11.41)	106 (8.611)	49 (10.606)	106 (8.249)	13 (11.017)	37 (7.613)	**7.90**
CcDEe	3660 (7.529)	165 (7.717)	87 (8.683)	22 (7.383)	97 (7.880)	40 (8.658)	109 (8.4825)	1 (0.8475)	28 (5.761)	**7.57**
ccDEE	529 (1.088)	22 (1.029)	14 (1.397)	2 (0.671)	11 (0.894)	3 (0.649)	21 (1.6342)	1 (0.8475)	10 (2.058)	**1.10**
Ccddee	270 (0.555)	17 (0.795)	5 (0.499)	2 (0.67)1	7 (0.569)	1 (0.216)	7 (0.545)	2 (1.695)	3 (0.617)	**0.56**
CCDEe	38 (0.078)	3 (0.140)	2 (0.200)	1 (0.336)	1 (0.081)	2 (0.433)	3 (0.234)	0	1 (0.206)	**0.09**
ccddEe	62 (0.128)	1 (0.047)	1 (0.100)	1 (0.336)	0	0	2 (0.156)	0	2 (0.412)	**0.12**
CCddee	6 (0.0123)	0.000	0.000	0.000	0.000	0.000	10 (0.7782)	0.000	0.000	**0.03**
CcDEE	4 (0.0082)	0.000	0.000	0.000	0.000	0.000	0.000	0.000	0.000	**0.01**
CcddEe	3 (0.0062)	0.000	0.000	0.000	0.000	0.000	0.000	0.000	0.000	**0.005**
ccddEE	2 (0.0041)	0.000	0.000	0.000	0.000	0.000	0.000	0.000	0.000	**0.004**

**Table 2:** Kell, MNS, Duffy, and Kidd blood group frequencies (%) compared with other ethnic groups.

Phenotypes	Our donors (%) (*N*)	Caucasians [16]	Blacks [16]
K+k−	0.19 (**1**)	0.2	Rare
K+k+	9.16 (**47**)	8.8	2
K−k+	90.64 (**465**)	91	98
S+s−	8.77 (**45**)	11	3
S+s+	34.31 (**176**)	44	28
S−s+	56.92 (**292**)	45	69
Fy (a+ b−)	19.30 (**99**)	17	9
Fy (a+ b+)	27.88 (**143**)	49	1
Fy (a− b+)	41.72 (**214**)	34	22
Fy (a− b−)	11.11 (**57**)	Very rare	68
Jk (a+ b−)	34.70 (**178**)	28	57
Jk (a+ b+)	49.51 (**254**)	49	34
Jk (a− b+)	15.79 (**81**)	23	9
Jk (a− b−)	0	Very rare	Very rare
